# SEEG4D: a tool for 4D visualization of stereoelectroencephalography data

**DOI:** 10.3389/fninf.2024.1465231

**Published:** 2024-09-03

**Authors:** James L. Evans, Matthew T. Bramlet, Connor Davey, Eliot Bethke, Aaron T. Anderson, Graham Huesmann, Yogatheesan Varatharajah, Andres Maldonado, Jennifer R. Amos, Bradley P. Sutton

**Affiliations:** ^1^Department of Bioengineering, University of Illinois Urbana-Champaign, Urbana, IL, United States; ^2^Beckman Institute for Advanced Science and Technology, University of Illinois Urbana-Champaign, Urbana, IL, United States; ^3^University of Illinois College of Medicine, Peoria, IL, United States; ^4^Jump Trading Simulation and Education Center, Peoria, IL, United States; ^5^Department of Neurology, Carle Foundation Hospital, Urbana, IL, United States; ^6^Department of Molecular and Integrative Physiology, University of Illinois Urbana-Champaign, Urbana, IL, United States; ^7^Carle Illinois College of Medicine, University of Illinois Urbana-Champaign, Urbana, IL, United States; ^8^Department of Computer Science and Engineering, University of Minnesota, Minneapolis, MN, United States; ^9^Department of Neurosurgery, OSF Healthcare, Peoria, IL, United States

**Keywords:** stereoelectroencephalography, SEEG, virtual reality, presurgical planning, epilepsy, visualization tools

## Abstract

Epilepsy is a prevalent and serious neurological condition which impacts millions of people worldwide. Stereoelectroencephalography (sEEG) is used in cases of drug resistant epilepsy to aid in surgical resection planning due to its high spatial resolution and ability to visualize seizure onset zones. For accurate localization of the seizure focus, sEEG studies combine pre-implantation magnetic resonance imaging, post-implant computed tomography to visualize electrodes, and temporally recorded sEEG electrophysiological data. Many tools exist to assist in merging multimodal spatial information; however, few allow for an integrated spatiotemporal view of the electrical activity. In the current work, we present SEEG4D, an automated tool to merge spatial and temporal data into a complete, four-dimensional virtual reality (VR) object with temporal electrophysiology that enables the simultaneous viewing of anatomy and seizure activity for seizure localization and presurgical planning. We developed an automated, containerized pipeline to segment tissues and electrode contacts. Contacts are aligned with electrical activity and then animated based on relative power. SEEG4D generates models which can be loaded into VR platforms for viewing and planning with the surgical team. Automated contact segmentation locations are within 1 mm of trained raters and models generated show signal propagation along electrodes. Critically, spatial–temporal information communicated through our models in a VR space have potential to enhance sEEG pre-surgical planning.

## 1 Introduction

Epilepsy is a chronic neurological condition affecting more than 50 million people worldwide. Epilepsy is characterized by recurrent, spontaneous seizures and is defined as two unprovoked seizures occurring more than 24 h apart, an unprovoked seizure if the risk of recurrence is high, or a diagnosis of an epilepsy syndrome (Fisher et al., 2014; Thijs et al., 2019). EEG recordings and physical behaviors clearly show how seizures produce strong electrical activity and spread throughout other areas of the brain. The exact pathophysiology producing the seizures (e.g., neurotransmitters, structural abnormalities, environmental factors), occurring at the seizure onset zone (SOZ), and how the electrical signals spread throughout the brain is not well understood. Identification of the SOZ is critical for treatment, particularly for surgical interventions. Imperfect identification of the SOZ renders imperfect treatments, which leads to continued seizures, additional surgical treatments, and overall reduction in quality of life (Andrews et al., 2020; Paulo et al., 2022).

Approximately 30–40% of patients who are diagnosed with epilepsy have symptoms which are not fully controlled by currently available antiepileptic medications, a condition known as drug-resistant epilepsy (DRE) (Kalilani et al., 2018). Such patients are at an increased risk of serious adverse effects resulting in significant degradation of their quality of life or premature death (Mula and Cock, 2015). For these patients, an effective treatment is a surgical resection of the area in the brain triggering the seizures, the SOZ (Ryvlin et al., 2014; Andrews et al., 2020). The goal of resective surgery planning is to outline the epileptogenic zone for an accurate surgery so that the patient can achieve seizure freedom (Ryvlin et al., 2014; Andrews et al., 2020). While the procedure is not risk free, cognition, behavior and quality of life can improve after resective surgery and it has proven to be an effective procedure (Ryvlin et al., 2014).

Determining the SOZ is often a difficult task because of the lack of morphological identifying characteristics distinguishable from healthy tissue in standard medical imaging evaluations (Ryvlin et al., 2014; Minkin et al., 2019). Localizing the SOZ typically involves a multi-modal approach combining various imaging modalities, such as magnetic resonance imaging (MRI), functional MRI (fMRI), computed tomography (CT), positron emission tomography (PET), magnetoencephalography (MEG), and electrophysiology using electroencephalography (EEG), along with neuropsychological testing and Wada testing (van Mierlo et al., 2020; Kakinuma et al., 2022; Bearden et al., 2023). A more invasive process, stereoelectroencephalography (sEEG), is used to obtain precise recordings from depth electrodes to identify SOZs that are deep in the brain or difficult to localize (Gonzalez-Martinez et al., 2014). In sEEG, neurosurgeons place electrodes into the brain, penetrating deep into the tissue, targeting regions that are suspected of being the SOZ to provide highly localized recordings in a 3D space to identify and confirm the seizure initiation site (Bartolomei et al., 2017). Electrode trajectories are often manually computed, but tools are being developed to assist with planning (De Momi et al., 2014).

Currently, epileptologists and neurosurgeons manually review the 1D sEEG recordings with the 2D multiplanar views of the 3D imaging data to localize the SOZ and epileptogenic activity (Hassan et al., 2020). Their goal is to construct a mental model of the patient’s specific anatomy when preparing for resective surgery (Minkin et al., 2019). This multimodal information is challenging for experts to mentally combine and extract actionable data (Lyuksemburg et al., 2023). Better mental representations of anatomy can be created from directly interacting with the 3D models as opposed to 1D and 2D views of the multimodal data (Guillot et al., 2007; Wu et al., 2010; Mattus et al., 2022). 3D models have proven useful for navigating through patient-specific anatomy in planning epilepsy surgery for both the surgeons and for patient education due to the integrated visualization of the complex multimodal data (Minkin et al., 2019; Phan et al., 2022). VR technologies can enable an interactive view of complex 3D models and have been used in other complex resection cases where they have demonstrated improvements in the operative experience for the surgeon (Quero et al., 2019; Louis et al., 2021). The seizure activity from the sEEG recordings creates even more complex data that are 4D, with 3 spatial dimensions and changes over time. In the current work, we further merge the clinical dataset from a sEEG study into a unified model for viewing anatomy and dynamic electrophysiological data in a 4D VR presurgical planning platform. This tool will enable surgeons to focus their attention and expertise on patient-specific details directly relevant to the surgery.

Several toolboxes have been developed to lessen the challenges involved with merging multimodal spatial information from sEEG studies (Armin Vosoughi et al., 2022). Some of these tools automate critical image information steps, such as isolating electrode contacts or making predictions about the SOZ or the epileptogenic zones on patient specific anatomy. A few examples include sEEG Assistant (Narizzano et al., 2017), which is a set of tools built as a 3D Slicer^1^ extension (Fedorov et al., 2012), and Epitools (Medina Villalon et al., 2018), which also uses Freesurfer (Dale et al., 1999) pial surfaces, or LeGUI (Davis et al., 2021). Many 2D sEEG visualization tools, such as Brainquake (Cai et al., 2021), opt to highlight or enlarge electrode contacts to indicate some degree of epileptogenicity while other software packages, like MNE-Python, project the data onto brain tissue and predict a SOZ in the brain based on the sEEG recordings (Gramfort et al., 2013; Cai et al., 2021). Several of the tools note if the contact predominately resides in gray matter or white matter, as tissue type can impact some of the computations made to analyze activity (Arnulfo et al., 2015). Some of the packages listed here require a brain atlas or a set of standard naming conventions which is not the case for all clinically acquired sEEG data sets. However, most of these tools are not maintained and are reliant on outdated dependencies that do not work with modern workstations as noted by Armin Vosoughi et al. (2022). Software such as EpiNav (CMIC, UCL, London, United Kingdom) (Vakharia et al., 2019) or CNSprojects^2^ are available in a limited manner and merge anatomical data with sEEG biomarkers. The virtual epileptic patient (Makhalova et al., 2022) based on Virtual Brain (Sanz-Leon et al., 2015) can take sEEG data and generate simulations of patients seizures, compute the SOZ and display a glass brain model and waveforms of the epileptic data. All these visualizations *present their models confined to a 2D display* requiring the surgeon to mentally extrapolate the data to 3D space and merge this activity data with the patient’s 3D anatomy for diagnosis and surgical planning. These tools do not resolve challenges with taking data presented in a 2D format and extrapolating it to generate 3D mental representations of the surgical case. SyncAR is an augmented reality and virtual reality platform which works with the data from surgical devices and uses VR for surgical planning and augmented reality to navigate during the resection procedure (Louis et al., 2021). SyncAR’s usage highlights a need for temporally dynamic visualization tools for sEEG evaluation and resection planning (Louis et al., 2021).

In this work, we propose SEEG4D, an open-source tool which presents the pre-implant MRI, post-implant CT, and dynamic sEEG data as a *4D dynamic model for use in a virtual reality (VR) presurgical planning environment* as opposed to the traditional 2D environment. By animating the time series data onto the electrode contacts in VR, we enable neurosurgeons to view the common components of the clinical data but in a platform that integrates spatial information with dynamic seizure activity to supplement the traditional resective surgery workflow. Users can virtually explore the 3D brain tissue, see electrode activation over time, and make surgical plans accordingly. 3D model use in pre-surgical planning capitalizes on this impact by creating improved mental representations of patient-specific anatomy through a personalized medicine approach (Wu et al., 2010). Previous research has shown that developing 3D VR models of patient anatomy has the potential to assist surgeons in presurgical planning and may reduce complications (Herfarth et al., 2002; Oldhafer et al., 2009; Chen et al., 2010; Quero et al., 2019). Further, situational awareness research analyzing expert performance over novice performance indicates improved mental models of pre-surgical anatomy are characteristic of the expert performer by shifting the mental burden from working memory to long term memory (Maan et al., 2012; Sadideen et al., 2013; Robertson et al., 2024). Additionally, VR has been shown to provide information which may alter the surgical approach (Quero et al., 2019; Mahajan et al., 2021; Robertson et al., 2024). SEEG4D seeks to automatically generate dynamic 4D models to supplement the presurgical workflow and enable VR-based presurgical planning. To our knowledge, SEEG4D is the first time that animated 3D VR models of electrical activity have been automatically generated and used for epilepsy pre-surgical planning.

## 2 Materials and methods

### 2.1 Software overview

**Figure 1:**
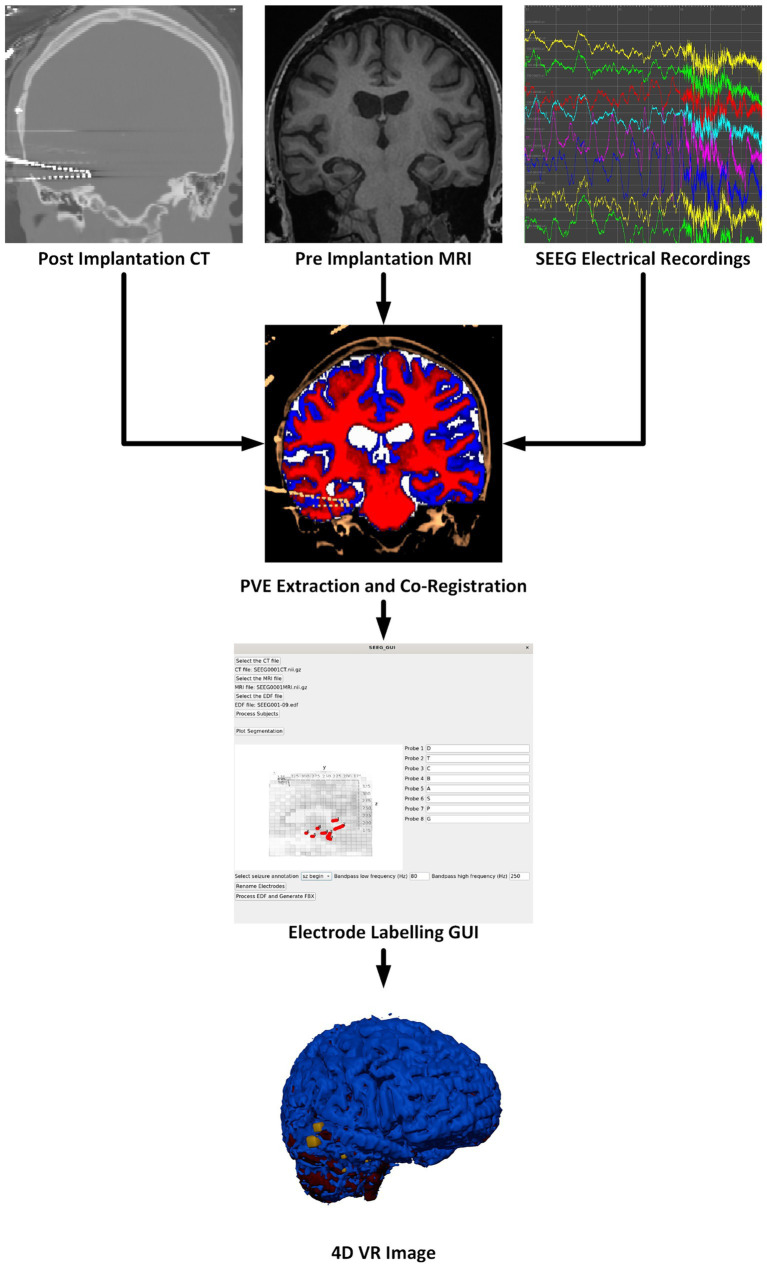
Overview of the software pipeline outlining input of CT, MRI, and sEEG data (as EDF files) through the GUI to create a VR image of the sEEG activity with the chosen bandwidth and window width. Labeling electrodes in the GUI with the sEEG planning map is the only manual step.

Our software package is split into two components. The first component is a Python-based GUI to handle user preferences, inputs, and provide status updates. The second component is a Docker container to perform the neuroimaging processing steps and generate VR-ready models. These components automatically interconnect and interact; users of the software need only install Docker and the bare minimum requirements to run the Python graphical user interface (GUI). Containerization of critical software components enables easier use and reproducibility of medical imaging software technologies (Matelsky et al., 2018). Key software included in our container is: Python 3.8 for FSL 6.0.5.1, Python 3.9 for MNE-Python 1.6.1 using nibabel 5.2.1 with scikit-image 0.22.0, Python 3.10 for Blender 4.0.0 (Python Software Foundation, https://www.python.org/) (Blender Foundation, https://www.blender.org/) (Woolrich et al., 2009; Gramfort et al., 2013; van der Walt et al., 2014; Brett, 2024). An overview of the multimodal image processing pipeline is shown in Figure 1.

There are many steps to processing sEEG data and merging the electrophysiology information into the patient’s 3D anatomical data. These steps are outlined in the flow chart in Figure 1 and briefly described here. More details are given in the following sections. The pre-implantation MRI is registered to the post-implantation CT which serves as the working space for anatomical images. The image processing steps, briefly, include: Segmentation of the pre-implantation MRI data into gray matter, white matter, cerebrospinal fluid (CSF), and any other regions of interest. Next, the electrode locations must be extracted, and the different electrode contacts must be merged into multi-contact electrodes. The naming of the electrodes is performed in the main GUI where the user associates the electrode names from the sEEG data and selects the corresponding segmented electrode in consultation with the sEEG implantation planning map. This is the only processing step which requires manual input as there is not a universal naming convention for implanted electrodes. sEEG data must be processed to identify the seizure timing and filtered according to a powerline noise notch filter combined with a user-selected bandpass filter design. By default, SEEG4D uses a frequency band of 80–250 HZ as this band is commonly used for the detection of high frequency oscillations which are correlated with epileptogenic activity (Remakanthakurup Sindhu et al., 2020). Electrode activity is then converted to an average windowed power. All 3D imaging processes and the SEEG processing are handled in the container, along with the Blender processes to generate the VR-ready 4D model as output. All code and containers are available on https://github.com/mrfil/SEEG4D.

### 2.2 3D image processing

**Figure 2:**
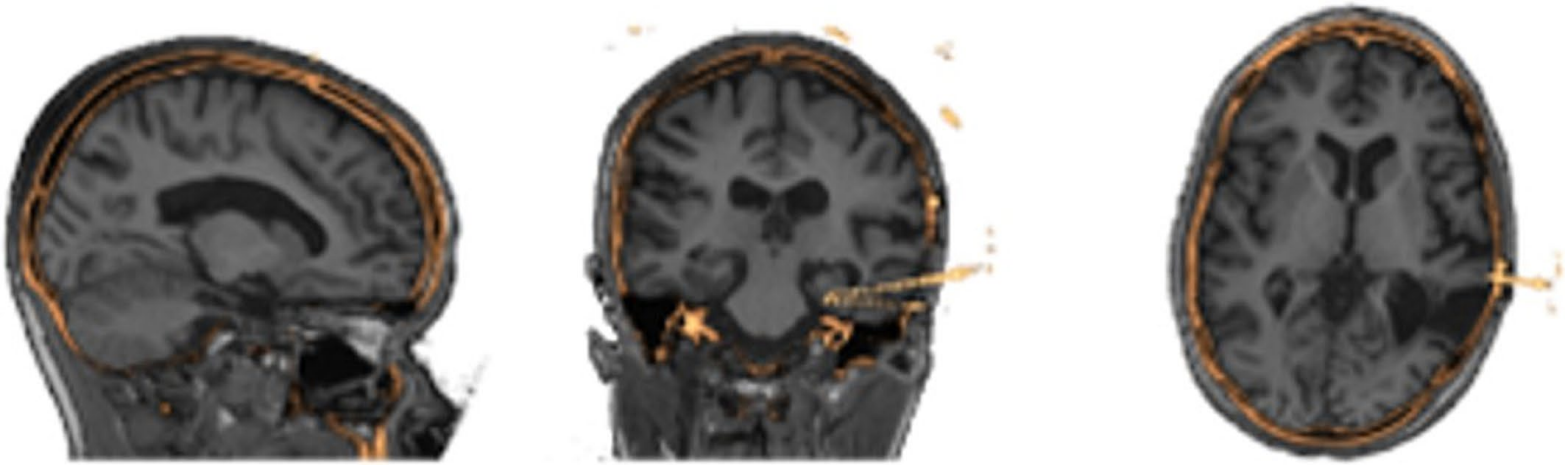
Results of image processing and registration as seen in three different views from the same patients’ MRI (grayscale) to their processed CT (gold) showing alignment of the two image spaces.

Image processing is done automatically using pre-existing neuroimaging software packages and customized python scripts installed inside the Docker container. A brain mask is generated using the FSL Brain Extraction Tool (bet) on the pre-implantation T1-weighted MRI (Smith, 2002). FSL FAST is used to segment the brain into gray, white, cerebrospinal fluid (Zhang et al., 2001). Registration between the CT and brain extracted MRI is performed by estimating a rigid body (6 DOF) transformation between the CT and MRI image using a mutual information cost function and FSL FLIRT (Jenkinson and Smith, 2001; Jenkinson et al., 2002). We apply the estimated registration to the brain mask and tissue type maps from MRI to put all MRI information into the CT space, with a nearest neighbor interpolation as shown in Figure 2. We erode the registered MRI mask three times so that there is little to no remaining overlap with the skull on the resulting registered mask. All images in the CT space are further resampled into 1 mm isotropic space and flipped, if necessary to align imaging space left/right to the future VR space left/right, to assist in the creation of 3D objects. SEEG contacts on the CT images are isolated from the skull by first applying the registered brain mask, followed by a threshold at the 99.5^th^ percentile to leave only voxels containing metal and metal artifacts. Through iterating, we found this chosen threshold removes the most noise, skull and scanner artifacts, and reduced contact blur (streaking artifact) without deleting contacts. A median filter with a sphere kernel of 0.5 mm is applied to the thresholded image to reduce contact streaking. Then, the filtered image is converted to isotropic space and a 40th percentile threshold is applied to remove interpolation artifacts. We note that our CT data did not have metal artifact reduction enabled in the acquisition leading to significant metal artifacts associated with the electrodes.

### 2.3 Electrode contact segmentation and aggregation

With the contacts isolated, SEEG4D locates the position of the contacts in space using a custom automated algorithm written in Python. A representative contact, which is a 3 mm x 3 mm x 3 mm isotropic voxel cube, was manually created to act as a template for future processing steps. Isolated voxels are treated as outliers and removed.

**Figure 3:**
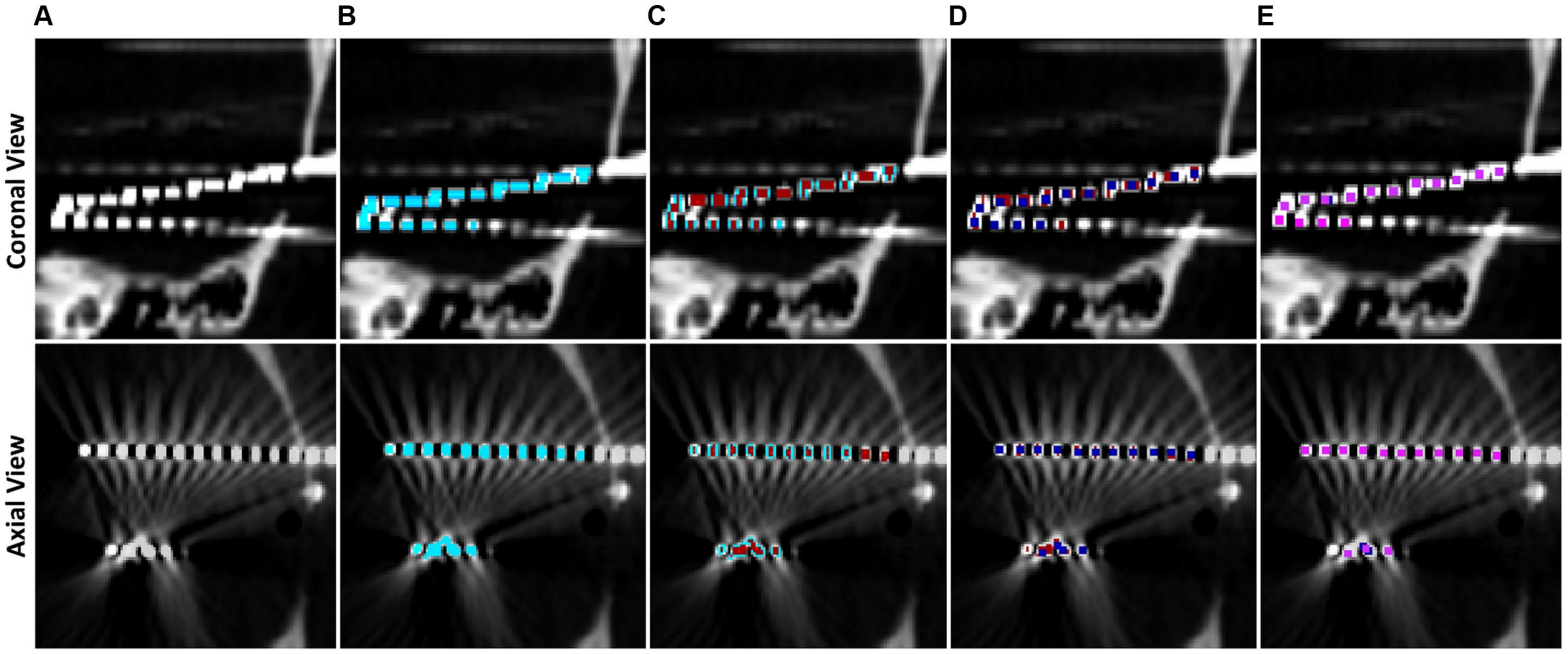
Step by step example of electrode contact segmentation and representative replacement. The top row is a cropped coronal slice while the bottom row is a cropped axial slice. **(A)** Base CT image, note that the ends of two electrodes have blended on the imaging and there is streaking artifact connecting two contacts. **(B)** Post masking, filtered, and thresholded electrode contact mask overlaid in blue. **(C)** Electrode contact mask after first pass of erosion in red, note that the ends of the electrodes have separated. **(D)** Second pass of erosion in dark blue. Contacts connected by streaking artifact have separated. **(E)** Final pass of the algorithm in pink. All contacts have been replaced by the representative contact by now.

Voxels are classified into contacts by grouping neighboring, non-diagonal voxels together recursively until every non-zero voxel has been grouped into a contact. We note that, due to streaking artifacts, this may group voxels from separate contacts together. Preventing the contacts from acquiring diagonal voxels helps prevent contacts which almost touch from clumping together. To further isolate contacts, we iteratively apply 1D erosions to electrode contacts until they are smaller than or the same size as the representative electrode via the following procedure, which is motivated by thinning connecting regions between contacts: If the contact is wider along the z direction than the representative, an x-directional erosion is applied; if the contact is wider along the x direction than the representative, a y-directional erosion is applied; if the contact is wider along the y direction than the representative, an x-directional erosion is applied. Once an electrode has been eroded to be smaller than half the size of the representative, it is replaced by the representative contact by aligning the midpoint of the representative to the replaced contact. Grouping and erosion algorithms run repeatedly on the entire image until all contacts have been replaced by the representative. This electrode segmentation process is demonstrated in Figure 3.

Electrode contacts can blur together due to scanner artifacts and orientation of electrodes in the scanner, so a contact-by-contact erosion method is preferred to separate the contacts and preserve spatial location of the contacts. Further, this individualized erosion approach works even with the difficult arrangements of electrodes that are not aligned with a main axis of the image, i.e., diagonal electrodes such as in Figure 3. Contact midpoints are saved and used to label them and orient them in space.

Collections of contacts to form electrodes are built from the midpoints of contacts by computing the outermost electrode contact and finding the closest contact and treating the pair as an electrode. The next contact within a search distance of 15 mm, and that does not deviate more than 20° from the second most recently added contact, is added to the electrode. Through iterative testing, we found 20° accounts for some bending along the electrode without merging parallel electrodes. This process repeats until all contacts have been classified into electrodes such as in Figure 3.

**Figure 4:**
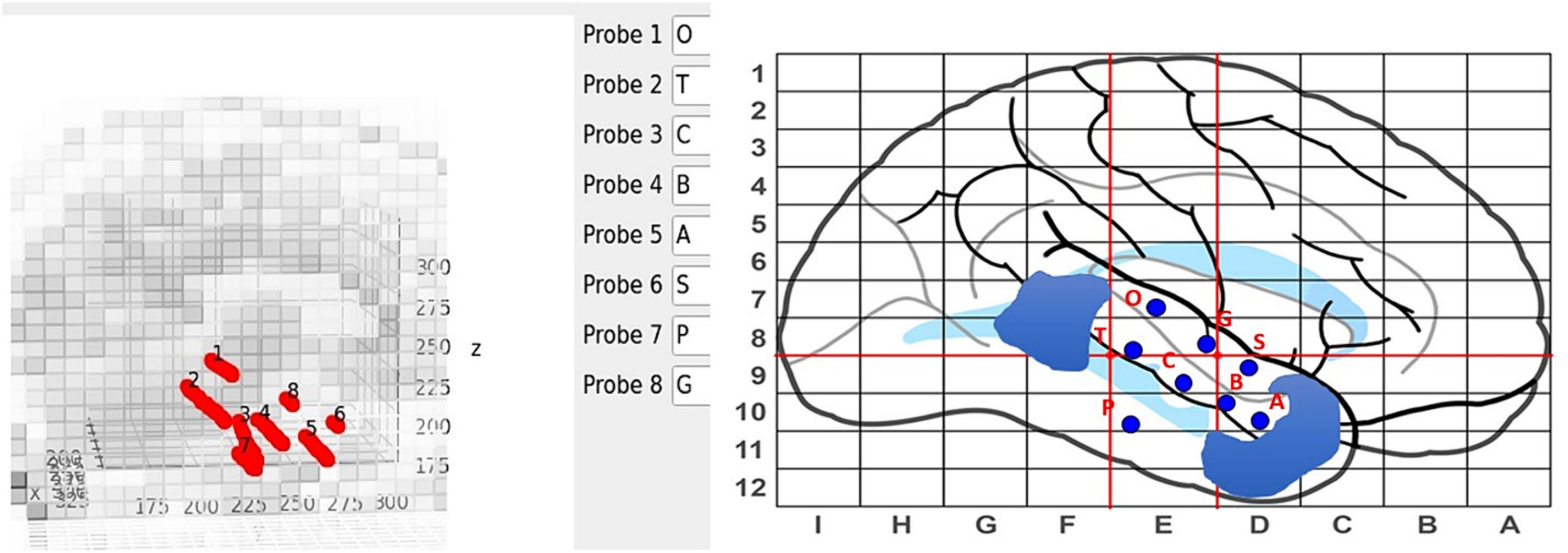
Labeling interface on the main GUI (left) with the sEEG planning map and naming scheme (right). Blue regions on the sEEG planning map indicate cavities.

Electrodes are manually labeled using the main GUI, see Figure 4, but contact numbering is done automatically. Axial and sagittal MRI slices are plotted along with the electrodes in a rotatable, zoomable interface. The center of the brain is computed and contacts along an electrode are labeled inner-most to out-most (e.g., A1 is electrode A contact 1 and is the contact at the end of the electrode) following our clinical site’s naming conventions.

### 2.4 sEEG data processing

Electrical data from the sEEG electrodes is provided to the software as EDF files. These files are loaded, through the Docker container, into MNE Python for signal processing (Gramfort et al., 2013). In the main GUI, a dropdown box is populated with the event flags in the EDF file where the user can select the flag belonging to the electrical activity of interest, such as a particular seizure. In this paper, we use events that were clinically marked as seizure start flags. By default, the signal is cropped around the chosen event with a two-second window on either side, creating a four-second clip in total for our 4D visualization. Data is notch filtered to remove power line noise and then any bandpass filters chosen are applied. By default, an 80 Hz-250 Hz windowed finite impulse response filter is used as this filter band is commonly used to identify high-frequency oscillations for SOZ localization (Remakanthakurup Sindhu et al., 2020).

### 2.5 VR model generation

Now that the SEEG contacts have been automatically segmented, named, and labeled with corresponding electrical data, SEEG4D generates VR models using Blender’s Python scripting capabilities. SEEG contacts, gray matter, white matter, and cerebral spinal fluid are each converted to object files using Scikit-Image’s implementation of the Lewiner Marching Cubes algorithm (Lewiner et al., 2003; van der Walt et al., 2014). Our sampling rate in the EDF files is approximately 1KHz, and we chose to make 24 ms wide long frames. Meaning that each second of the animation contains 24 ms of data. This turns a 4-s-long EDF clip into a 167-s-long animation. We compute the power over our sliding window by:


$$P_{x}={\left\|\frac{1}{N}\overset{N}{\underset{n=1}{\sum }}=10\;{\left(x\left[n\right]\right)}^{2}\right\|}_{2}$$

Where *N* is our sliding window length (*N* = 24). Min-max scaling is applied to the power data, across all electrodes by subtracting the minimum power and dividing by the range of power across all windows and contacts.

Electrode contacts are animated by evenly scaling their size at the frame being animated, where the maximum size is 6 cm, to make the difference in power between electrodes more apparent. Visually larger contacts have proportionally more power at that frame than smaller contacts. A timeline was manually created using blender to indicate time along the animation. This timeline includes markers for every second of the electrical data and a red marker indicating the marked seizure start. Once all contacts have been animated, and the brain segmentations and timeline have been loaded into the Blender model, the model is saved as both an FBX file and a GLTF 2.0 file which can be loaded into VR.

**Figure 5:**
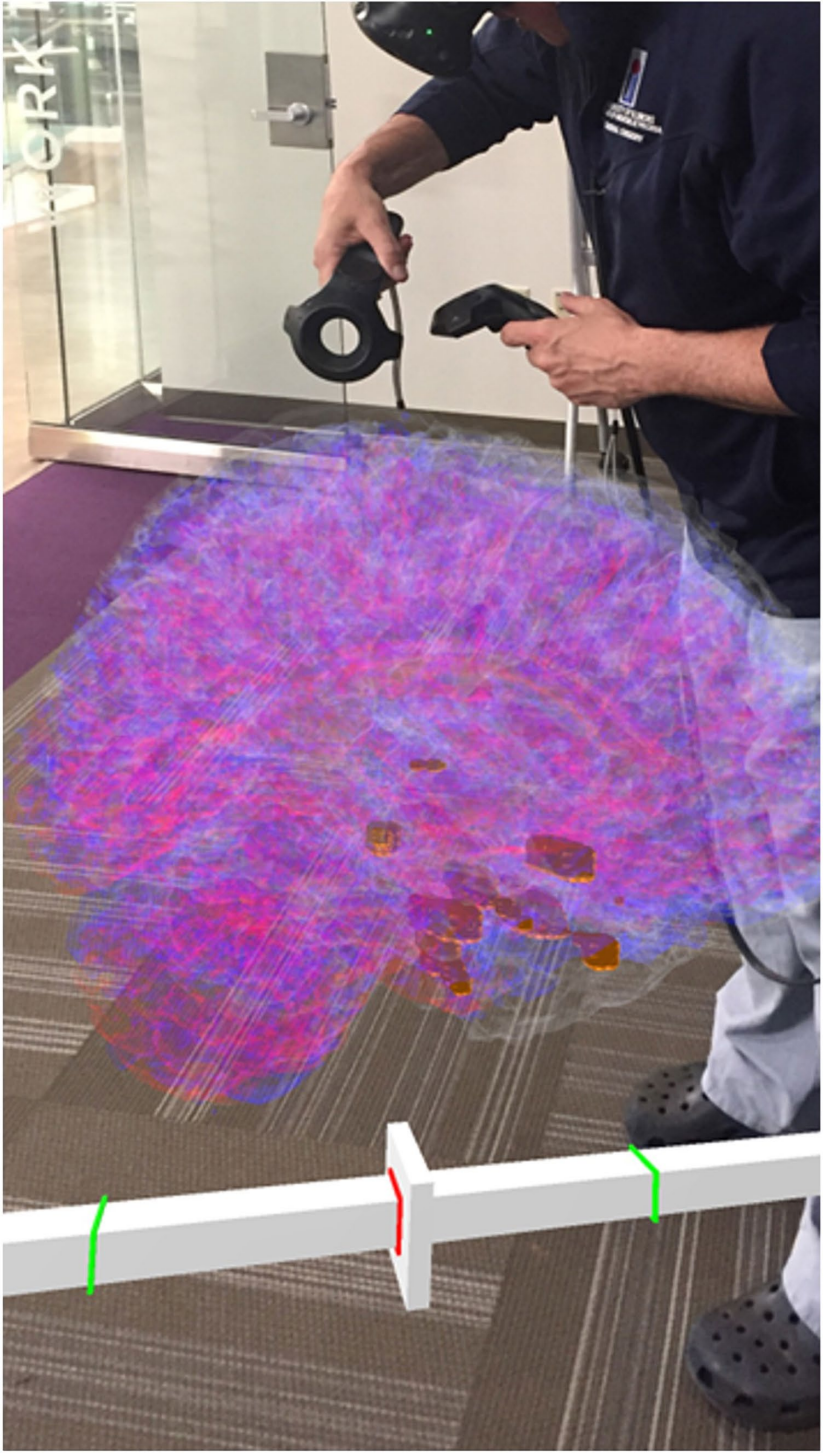
A clinician interacting with the 4D SEEG4D generated model in virtual reality. Electrodes are shown in gold and their size indicates relative power, whereas electrodes with a higher power are larger. A timeline appears underneath the brain to indicate the currently viewed timing relative to the marked seizure event (red line).

**Figure 6:**
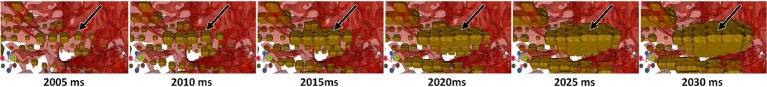
4D signal propagation along an electrode at time points varying by 5 ms during a seizure. Contacts along this electrode become larger in sequence. Timepoints were extracted from Blender.

Our SEEG4D creates the 4D FBX model and converts other supplementary documents into pdf versions for loading into the VR software, including the electrode surgical map and the electrode recording data graphs. Visualizing the data and model requires a VR platform for viewing and interacting with the generated assets. Many options exist for this. In this study, we imported the data into Enduvo,^3^ as shown in Figure 5, and Blender, as shown in Figure 6.

### 2.6 Test patient data

To test the capabilities of SEEG4D and the automated electrode location labeling, data from 3 temporal lobe epilepsy patients undergoing clinical epilepsy monitoring at the OSF Saint Francis Medical Center, Peoria, Illinois, were run through our software in a fully automated processing, except for the manual selection of the electrode names in the GUI based on the sEEG planning map. DIXI sEEG electrodes (DIXI Medical) 0.8 mm in diameter and 2 mm apart were sampled at 1 KHz during monitoring. Deindentifiedatient data was acquired through OSF HealthCare under an IRB approved by University of Illinois College of Medicine at Peoria IRB.

To test the accuracy of our automated electrode-contact labeling process, we had two trained anatomists with a combined 5 years of segmentation experience label the electrode contacts manually. Our trained manual raters used 3D Slicer to mark the location of the electrode contacts from all electrodes from the 1 mm isotropic CT image to identify the recording locations (Fedorov et al., 2012). In the event a rater marked a position in between voxels, their marker was rounded to the nearest voxel. This manually labeled electrode contact center was compared to the corresponding automated electrode contact’s center and to the positions from the other rater.

## 3 Results

SEEG4D processed 3 cases on a machine running Ubuntu 22.04.4 LTS with 96 GB of memory, an Intel^®^ Xeon^®^ Gold 6,254 CPU @ 3.10GHz x 72, and three NVIDIA Quadro RTX 8000. Brain extraction and electrode segmentation took approximately 50 min per patient. Processing the sEEG data took approximately 5 s per patient while animating the data with Blender took approximately 30 s leading to a total runtime of under an hour per patient.

### 3.1 Electrode segmentation validation

After processing our 3 cases, SEEG4D identified 271 contacts in total. From visual inspection, we found that the contacts had good concurrence with the ground truth CT data. The average distance between these automatically identified coordinates and the manually labeled coordinates was variable per case, but as shown in Table 1 the electrode localization algorithm was generally closer to the raters than the raters were to each other indicating good concurrence with the ground truth position. As an example, for case SEEG1, our algorithm was an average of 0.85 mm away with a standard deviation of 0.68 mm from rater 1’s midpoints and an average of 0.71 ± 0.74 mm from rater 2’s midpoints while the raters were an average of 0.94 ± 0.52 mm from one another. We note that 94.8 and 85.2% of the contacts automatically identified were within 1 voxel of rater 1 and rater 2, respectively. Additionally, 89.7% of the raters’ contacts were within 1 voxel of each other. We define a contact that the algorithm ‘missed’ as a contact residing in brain tissue that was not labeled by the software. Notably, contacts in the skull or outside the head are not counted as ‘missed’. Shown in Table 2, for SEEG1, there were 2 missed contacts of 116 (1.7%); for SEEG2, there were 3 missed contacts of 91 (3.3%), and for SEEG3 there were 0 missed contacts of 71 (0%). Of these 5 missed contacts, all were within 5 voxels of the edge of the cortex and were either masked out or eroded during imaging preprocessing. Table 2 also shows that 82, 93, and 80% of segmented contacts were within 1 voxel for cases SEEG1, SEEG2, and SEEG3. Segmented contacts that were more than 2 voxels away were due to blurring and streaking on the contacts from the CT causing the contacts to appear larger, and when erosions were applied it offset the midpoint of the contact.

**Table 1:** Quantitative analysis of electrode localization algorithm showing the average distance and the standard deviation between the raters and algorithm per patient case as well as the distance between each rater.

Average distance per contact (mm)					
SEEG1		SEEG2		SEEG3	
Rater 1	Rater 2	Rater 1	Rater 2	Rater 1	Rater 2
0.85 ± 0.68	0.71 ± 0.74	0.76 ± 0.60	1.0 ± 0.57	0.61 ± 0.62	1.0 ± 0.71

Raters distance from each other (mm)		
SEEG1	SEEG2	SEEG3
0.94 ± 0.52	0.93 ± 0.40	0.98 ± 0.49

The top table is the difference between the automated algorithm and each of the 2 manual raters. The bottom table is the differences between the 2 manual raters.

**Table 2:** Voxel distance of algorithmically determined contact midpoints to averaged rater-labeled midpoints.

Voxel distance of algorithm to rater average			
Distance	SEEG1	SEEG2	SEEG3
N < = 1 Voxel	92	82	57
1 < N < = 2 Voxels	17	6	14
2 < N < = 3 Voxels	3	0	0
Missed	2	3	0
Number of contacts (*N*)	112	88	71

82, 93, and 80% of segmented contacts were within 1 voxel for cases SEEG1, SEEG2, and SEEG3, respectively. five contacts were missed by the algorithm in total, all of which were within 5 voxels of the edge of the brain.

## 4 Discussion

We have developed a tool, SEEG4D, which merges pre-implant MRI, post-implant CT, and SEEG data to create a 3D model of an sEEG case with time series data mapped onto the contacts. This enables the automated creation of digital assets for use in a 4D VR surgical planning process to enable the clinical care team to localize the SOZ and plan surgical interventions.

### 4.1 Visualization analysis

Presurgical planning for resection of SOZ is a highly complex process involving multi-modal 2D and 1D (SEEG) medical data. When one considers that interpretation of this complex patient-specific data by one medical expert is then communicated to a different surgical expert to resect a specific SOZ in the brain, there is tremendous opportunity to improve the precision of shared mental models of the pathology.

This project was initiated to improve knowledge transfer of patient-specific, complex, multimodal information on the location and pathology of the SOZ from epileptologist to neurosurgeon. To achieve translational impact, automated tools were developed along with stereoscopic time-sequential 3D digital models. These were necessary to allow integration into a clinical workflow where time constraints prevent manual efforts of 4D model creation. We have successfully deployed our software package, enabled by the containerization of the algorithms, in the clinical environment for research purposes and ran cases for this study on the clinic computational hardware.

Preliminary qualitative feedback revealed that the clinical sEEG expert sees tremendous potential of SEEG4D to expedite review of the standard of care data by helping to merge multimodal information about a seizure to provide an improved understanding of the patient’s electrophysiological data. Our surgical expert indicated significant potential of SEEG4D to improve communication of the 3D location of the SOZ from epileptologist to surgeon. Our experts, combined, see this tool as a new framework for forming mental models to allow for more efficient yet robust discussion for each patient.

SEEG4D allows users to automatically animate the electrical data at electrode contacts over time. Since the timescale is slowed down, we see clear visual onset and propagation of signals between electrode contacts during a seizure, as shown in Figure 6 and Supplementary Video S1. This can facilitate understanding of propagation of the seizure and localization of SOZ. Incorporation of additional data into the visualization is straightforward, such as including white matter fiber pathways identified through diffusion tensor imaging to examine the relationship between the electrical signal propagation and tractography. Further work is required to understand the impact of increasing visual complexity of the visualized model on improving understanding of the patient case.

One of the limitations with SEEG4D is that it requires T1-weighted non-contrast MRI data. Additionally, our CT scanner was configured in a way which caused a substantial amount of metal artifacts at acquisition, leading to the automatic processing steps requiring higher thresholds and more aggressive erosion schemes. All cases processed for this study used DIXI electrodes and our tool is optimized based on these electrodes. It will be necessary to test SEEG4D against data from other clinical sites to ensure that these optimizations do not degrade cases that have little streaking or use other electrode manufacturers. Additionally, our clinical data did not use a standard naming convention for electrodes, so the software does not support loading of an atlas-based automatic naming scheme for electrodes.

While our clinicians have expressed qualitative feedback indicating that this tool would lead to a significant reduction in the time it takes to determine and understand a SOZ, quantitative analysis of this impact will be provided in a future study. To demonstrate quantitative impact on the clinical workflow, we will evaluate the efficacy of this model and quantify the reduction in mental load during the pre-surgical planning period for new cases. Additionally, the inclusion of source localization using automated SOZ localization algorithms to show the SOZ in the VR space could provide useful information to the clinical team.

## 5 Conclusion

We developed SEEG4D, a tool for automatically visualizing SEEG data with 4D virtual reality models for presurgical planning for epilepsy resection surgery. SEEG4D improves presurgical planning in epilepsy resection cases by automatically merging multimodal imaging data from MRI, CT, and sEEG recordings to produce dynamic 4D VR visualizations of seizure onset and propagation to facilitate the formation of an accurate mental model of the case. Our automated sEEG electrode contact detector was demonstrated to be accurate to within 1 mm of our ground truth raters. Models generated from SEEG4D provide an advantage over traditional sEEG models due to their interactive, 4D spatiotemporal nature. Our interactive models show signal propagation along electrodes and through local networks to additional recording sites. With this automated tool, epilepsy care teams may realize the potential of integrating dynamic sEEG data with VR for enhanced presurgical planning and the formation of shared mental models.

## Data Availability

The datasets presented in this article are not readily available because the software directly integrates with clinical data which may not be fully de-identified. Requests to access the datasets should be directed to Bradley Sutton, bsutton@illinois.edu.

## Footnotes

^1^ https://www.slicer.org

^2^ https://cnsprojects.nl/products/

^3^ https://enduvo.com/

## Supplementary material

Supplementary Videos are provided to help demonstrate the rich information that is present in the 4D models and VR environment for an epilepsy case. It is challenging to convey the breadth of information present in a 4D model using a 2D print format. Without being able to interact with the models and play the animations, sense of time, depth, and texture are impeded. To help alleviate this, we have included Supplementary Videos of the models and user interactions with them. In addition, our code and sample data are available on our GitHub at https://github.com/mrfil/SEEG4D, DOI: 10.5281/zenodo.12741316.
